# Implementation of a Commitment Machine for an Adaptive and Robust Expected Shortfall Estimation

**DOI:** 10.3389/frai.2021.732805

**Published:** 2021-08-31

**Authors:** Marco Bagnato, Anna Bottasso, Pier Giuseppe Giribone

**Affiliations:** ^1^ Data and AI, SoftJam, Genoa, Italy; ^2^ Department of Economics, University of Genoa, Genoa, Italy; ^3^ Financial Engineering and Data Mining, Banca CARIGE, Genoa, Italy

**Keywords:** expected shortfall, monte carlo methods, stochastic differential equation, bayesian vector autoregressive, dynamic neural networks, nonlinear auto-regressive networks, artificial intelligence, commitment machine

## Abstract

This study proposes a metaheuristic for the selection of models among different Expected Shortfall (ES) estimation methods. The proposed approach, denominated “Commitment Machine” (CM), has a strong focus on assets cross-correlation and allows to measure adaptively the ES, dynamically evaluating which is the most performing method through the minimization of a loss function. The CM algorithm compares four different ES estimation techniques which all take into account the interaction effects among assets: a Bayesian Vector autoregressive model, Stochastic Differential Equation (SDE) numerical schemes with Exponential Weighted Moving Average (EWMA), a Generalized AutoRegressive Conditional Heteroskedasticity (GARCH) volatility model and a hybrid method that integrates Dynamic Recurrent Neural Networks together with a Monte Carlo approach. The integration of traditional Monte Carlo approaches with Machine Learning technologies and the heterogeneity of dynamically selected methodologies lead to an improved estimation of the ES. The study describes the techniques adopted by the CM and the logic behind model selection; moreover, it provides a market application case of the proposed metaheuristic, by simulating an equally weighted multi-asset portfolio.

## Introduction

The cross correlation of assets and the time-varying behavior of conditional volatility are two important risk factors for an investment portfolio. Volatility tends to grow during periods of financial crisis, as shown for example by Ang and Chen (2002). This leads to periods of volatility clustering where conditional, short term volatility is very different from the long term unconditional one, with substantial effects on portfolio shocks that can be modeled through GARCH and EWMA volatility models. Under these conditions, an increase in correlation can intensify the effects of correlated shocks on portfolio losses. Therefore, the objective of this work is to create a Risk Management system able to estimate the coherent statistical measure of Expected Shortfall through the implementation of models that take into account the cross-correlation between assets and the trend of variance clustering over time; moreover, the study suggests a possible solution to the problem of selecting adaptively the most performing ES models.

In particular, the proposed solution consists in the implementation of a metaheuristic, called Commitment Machine (CM), that allows to choose, day by day, the ES estimation that best fits the financial market conditions.

Differently from our previous study (see Bagnato et al. (2021)), we decided to implement the Expected Shortfall risk measure rather than Value-at-Risk, because the former is always a coherent statistical measure of risk. In other words, only the ES satisfies the sub-additivity property: it is true that ES(X+Y)≤ES(X)+ES(Y) but it is not always verified that VaR(X+Y)≤VaR(X)+VaR(Y), where X and Y are random variables representing the returns of a portfolio (see Acerbi and Tasche (2002)). Indeed, the risk of two portfolios (or assets) together (i.e., X and Y) cannot be any worse than the sum of the two single risks: this is the well-known financial diversification principle. Although VaR is a less theoretically consistent measure than ES, it is worth noting that it continues to be a very popular risk measure among Financial Institutions because in the majority of cases the mathematical property is anyhow empirically verified.

Taking into account the important role of correlation, we have decided to employ four ES estimation models that are able to incorporate this fundamental factor in the simulation. As a result, we decided not to include in the CM the more traditional backward-looking methods, such as historical ES and parametric ES, but to adopt more dynamic techniques.

The four ES estimation techniques (see Table 1) implemented in the CM are:

• a Bayesian Vector AutoRegressive model (BVAR) with a prior distribution that simulates a stochastic behaviour for both the cross correlation among asset components and their volatility;
• a Euler-Maruyama SDE numerical scheme with a EWMA volatility and a Cholesky Decomposition for the correlation;
• a Euler-Maruyama SDE numerical scheme with a GARCH volatility and a Cholesky Decomposition for the correlation;
• a hybrid Monte Carlo method that uses the predictions of Non-linear AutoRegressive (NAR) networks as drift, a GARCH volatility and a Cholesky Decomposition for the correlation.

**TABLE 1:** Implemented models in the Commitment Machine.

Model	Volatility	Correlation
Bayesian Vector autoregressive model (BVAR)	a prior distribution	a prior distribution
Euler-Maruyama SDE numerical scheme	EWMA	Cholesky Decomposition
Euler-Maruyama SDE numerical scheme	GARCH	Cholesky Decomposition
Hybrid Monte Carlo method with NAR	GARCH	Cholesky Decomposition

The metaheuristic of the CM is able to evaluate the four approaches by calculating every day, for each of them, the value assumed by a loss function in the previous days. The model with the best performance is then chosen to estimate the ES of the following day. In our case, the loss function is equivalent to the sum of portfolio returns below the ES threshold, and it represents the effects of extreme events that are of greater magnitude than the expected value of losses below the ES threshold.

The CM overall performance and selection capacity have been assessed by analysing the losses with respect to the ES threshold and the comparative performances of the single methods when they are selected by the CM. The results clearly indicate that the CM is able to make an efficient selection among the various methods, by choosing ES thresholds that are less likely to be violated. The proposed Risk Management approach is extremely customizable. Indeed, thanks to the flexibility of the code written in MATLAB^®^, the CM approach can be used to select among a great variety of ES methods.

## Dataset and Portfolio Construction

For the purposes of the analysis, we built an equally weighted portfolio using four historical time series retrieved from the info-provider Bloomberg^®^. These series track four different indices representing three of the main asset classes available to investors (equities, bonds and gold). The components of these indices are representative of the investment choices of most financial intermediaries and allow us to represent a balanced portfolio:

• European Stock Index (SXXP Index): the Stoxx 600 index tracks the trend of large, mid and small cap stocks in 17 different European countries. With its 600 components, it allows to simulate a highly diversified equity portfolio across UK, Switzerland and the Eurozone.
• US Stock Index (RAY Index): The Ray Index includes 3,000 listed companies which represent 98% of the universe of US listed shares (in terms of market cap), allowing US stock markets to be incorporated into the portfolio.
• World Bond Index (Legatruh Index): the Bloomberg^®^ Barclays Global Aggregate Index collects investment grade debt listed on 24 markets, in both developed and emerging economies. The inclusion of this index allows to increase diversification by adding a second asset class distinct from the stock market and by minimizing the geographic risk.
• Gold (XAU USD currency): this series tracks the historical exchange rate between gold and the US dollar. Gold has traditionally been considered a safe-haven asset and its inclusion can offer significant diversification potential.

Since the European stock index is denominated in Euro, we have retrieved the historical Euro/Dollar exchange rates for the analysed period and used them to convert all the data into US dollars. In order to achieve a reasonable sample size, we decided to analyze the data of the daily closing prices for the period from 1st June 2000 to 30th September 2020. This time span contains a total of 5,305 market observations for each considered asset. The analysis requires the choice of a time window that allows to dynamically evaluate the evolution of the risk measures and of all other relevant variables (particularly the evolution of cross correlations in order to have a correct measurement of portfolio risk). This observation window (“rolling windows”) must be large enough to be statistically significant, but at the same time it should not be too wide in order to concretely capture the effects of relatively short-term shocks (for example the collapse and subsequent recovery of the markets due to the Covid-19 pandemic in the spring of 2020).

In order to balance the above-mentioned trade-off, in accordance with the practice used for this type of analysis, we decided to use a rolling window of 260 observations, which is equal to 1 year and is considered large enough for an overall analysis of market risk.

Table 2 shows the main descriptive statistics (minimum, maximum, median and the first four moments) for each historical time series. The various data are calculated on the entire history available without the use of rolling windows. All series show a high level of kurtosis and this seems to suggest a non-normal distribution for our data.

**TABLE 2:** Analysis of portfolio and components time series.

	Distributional and descriptive statistics of daily returns						
	Min	Max	Median	Average	St. Dev.	Kurtosis	Skewness
US equity	−11%	10%	0,026%	0,006%	1,21%	10,49	−0,22
EU equity	−12%	13%	0,061%	0,032%	1,44%	12,06	−0,12
Bond market	−2%	2%	0,024%	0,020%	0,16%	11,60	−0,41
Gold	−9%	11%	0,045%	0,042%	1,07%	9,19	−0,19
Portfolio	−7%	6%	0,04%	0,025%	0,65%	12,77	−0,27

In order to analyse data distribution, we performed a Kolmogorov-Smirnov test at 5% significance level that prompted us to reject the normality hypothesis for all four time series (*p* value ∼0 for all the series). The rejection of the normality hypothesis of our dataset prevents the adoption of the variance-covariance method in the ES calculation. Consequently, this approximated method of estimation has not been adopted in this study.

The second consideration deals with the most volatile among the indices i.e., the two equity indices. The matrix of daily correlations across the indices, reported in Table 3, shows that the greatest correlation is observed between the US stock index and the European stock index. The correlation between portfolio assets can be considered as a possible risk factor. The greater riskiness of the two equity indices has been confirmed by the Euler decomposition of portfolio risk (see Tasche (2008)), suggesting that more than 75% of the portfolio volatility is generated by the two most volatile equity indices. More specifically, the Euler decomposition attributes portfolio risk among different components as follows: 32.3% to the European stock index, 43% to the American stock index, 1.2% to the bond market index, 23.5% to the gold one. This calculation highlights how the correlation structure is by itself a risk factor, as it has the potential to amplify losses due to the most volatile indices in the portfolio.

**TABLE 3:** Cross correlation matrix of portfolio components.

Cross correlation matrix of portfolio components				
	EU equity	US equity	Bond market	Gold
EU Equity	1,00	0,51	−0,29	−0,01
US Equity	0,51	1,00	−0,18	0,002
Bond market	−0,29	−0,18	1,00	0,18
Gold	−0,01	0,002	0,18	1,00

Another important feature of the cross-correlations between assets is the opportunity to use them to build less procyclical models. Over the sample period, the two major negative events (the 2008 crisis and the Covid shock) came after a long period of positive equity market returns; in both cases, the value of the portfolio reached an all-time high just before the crisis. This is an obvious issue for Risk Management: the most common models of ES are strongly backward looking, with obvious negative effects when indices suddenly shift from growth to collapse. However, an analysis of the cross-correlations between assets can help to solve this problem: in both cases mentioned above, the correlation between the two equity indices started to rise before the onset of the crisis. In the case of the 2008 crisis, in the previous 2 years both the variance of each of the two indices and their covariance increased, while before the 2020 crisis the two variances were stable.

More generally, it is clear that a Risk Management model that includes the cross-correlation between assets among its input arguments can provide a more risk-sensitive estimate than a model that does not take these variables into account.

Leaving aside a more in-depth analysis of the patterns observed in 2008 and 2020 during the crises (which is not the aim of this study), it can however be assumed that a higher level of speculative investment in the markets leads to an increase in returns and therefore to a greater degree of vulnerability in case of a shock; in this scenario, the presence of diversified portfolios in the markets can lead to an increase in the risk of contagion between different assets, as experimentally shown by Cipriani et al. (2011), even when the same assets continue to have positive returns for a certain period.

Another point of view–from an econophysics perspective - expressed in particular by Sornette (2017), who analyzes the financial markets by using systems theory, sees financial crises as a “critical point” of a system, that happens due to the effects of the collective and increasingly correlated behaviour of several operators. In this sense, Sornette defines an “emergent cooperative behavior” which reaches its maximum in the moment immediately preceding the collapse of the market, leading to the outbreak of the crisis. More generally, as observed by Khandani and Lo (2007) in a case study on a sudden shock of the US hedge fund market in August 2007, in recent years the interconnection between different financial institutions (see also Agosto et al. (2020)) has led to an increase in systemic risk, which is also reflected in the cross-correlations between different financial instruments and assets.

The main point of this analysis, beyond the possible explanations, is that the cross correlation between the most volatile assets is a proxy of the overall risk and incorporating it into a Risk Management system seems to offer great modeling benefits. This is even more true to the extent that this risk takes the form of a sudden increase in market volatility (see Ahelegbey and Giudici (2020), Giudici et al. (2020)).

In this perspective, the inclusion in all our models of a stochastic component related to both correlation (a priori distribution for the Bayesian method, Cholesky decomposition for the Monte Carlo method) and volatility (EWMA and GARCH for the Monte Carlo method, a priori distribution for the Bayesian model) can be seen as a two-stage adaptation process: in the first stage, pre-crisis and forward looking, the Risk Management model collects information on the increase in correlations, and this information generates a more prudential approach to the extent that the correlation affects the simulated shocks. At this stage, systemic risk is rising, but the only proxy for such risk (besides the large returns) is correlation.

In the second phase, portfolio returns fall and the inclusion of models that consider short-term conditional volatility (Monte Carlo GARCH and in particular the EWMA version) accelerates the “adaptation” of the models to the new scenario. At this stage, a GARCH volatility representation attributes a greater weight on long-term volatility, and is therefore less conservative in a context where short-time volatility is higher than the long-term one. Hence, the Monte Carlo simulation with the GARCH method has been enriched with a non-linear autoregressive structure (i.e., a Neural AutoRegressive - NAR Network), which replaces the expected value of the returns in the drift of the stochastic differential equation. In this case, the model maintains a high degree of dynamism by giving the right consideration in terms of weight to the most recent components of the information set.

### Description of the Expected Shortfall Methodologies

After choosing the dataset, we have built a Risk Management system that can serve as a basis for the analyses. For the purpose of calculating the ES, four different methods have been implemented in order to take into account both sudden changes in variance (or, in other words, to distinguish between conditional and unconditional variance) and the effects of the cross-correlations.

### Bayesian Vector Autoregressive Model (Bayesian VAR)

Taking as a reference an econometric model written in the form:

y = Xβ+ ε ε∼N(0,Σ) (1)

Where y is the Txn matrix that collects the predicted returns for the n endogenous variables and *X* is the Txk matrix that collects the past returns. T is the length of the considered time series and k=n⋅p where p are the lags. The main parameters are the matrix of the coefficients *β* (whose dimension is kxn) and the nxn variance-covariance matrix of the errors Ʃ, which in this case is distributed according to a multivariate Normal and is used to generate the Txn error vector ε. The principle of Bayesian analysis consists in putting together the information that is available in advance on the distribution of these parameters (the so-called prior distribution) with the information that we can obtain from the data (i.e., the likelihood function). In this way it is possible to obtain a new probability function that considers both factors, the so-called posterior distribution. The essential step for putting together the prior distribution and the likelihood function is the Bayesian rule. For a vector of parameters θ and a dataset y, given the density function f (y | θ), the Bayesian rule can be expressed as:

$$\pi \left(\theta \text{|}y\right)=\frac{f\left(y\text{|}\theta \right)}{f\left(y\right)}\pi \left(\theta \right)$$ (2)

The formula states that, given y, the probability that the “true value” that the parameter vector is θ is equal to the likelihood function of the data multiplied by the a priori distribution of the vector of parameters *π* (θ) and divided by the density of the data f (y). This probability is expressed as the posterior distribution of *θ* given y, indicated as π(θ|y).

The vector of the parameters *θ* mentioned above is made up of two different elements: the vector of the coefficients *β* and the variance-covariance matrix of the errors Ʃ. For each of these elements, it is necessary to specify a prior probability distribution that allows - together with the likelihood function - to implement the “Bayesian rule.” One of the most widely used prior distribution is the “Minnesota prior” (see Dieppe et al. (2018)).

The Minnesota prior assumes that the variance-covariance matrix Ʃ is already known. Therefore, only the vector of the coefficients β remains to be estimated: for this purpose, it is necessary to identify the likelihood function of *β*, f (y| β), and a prior distribution π(β). The starting point is the likelihood function: Eq. 1 implies that y is distributed as a normal multivariate distribution with mean Yβ and variance-covariance matrix Ʃ. Various techniques can be employed in order to estimate the matrix Ʃ. With enough computational power it is possible to relax the hypothesis of the diagonality of the Ʃ matrix as described by Litterman (1986) and derive it from the variance-covariance matrix of a similar VAR model estimated via Ordinary Least Squares–OLS. Consequently, the maximum likelihood function can be written as:

$$f\left(y\text{|}\beta ,\Sigma \right)={\left(2\pi \right)}^{-\mathrm{nT}/2}|\Sigma {|}^{-\frac{1}{2}}\exp\left[-1/2\left(y-\bar{Y}\beta \right)\prime {\bar{\Sigma }}^{-1}\left(y-\bar{Y}\beta \right)\right]$$ (3)

Where n is the number of endogenous variables in the considered model.

The notation can be simplified by collecting under the α parameters the terms that do not depend on *β*:

$$f\left(y\text{|}\beta ,\Sigma \right)=\mathrm{\alpha exp}\left[-1/2\left(y-\bar{Y}\beta \right)\prime {\bar{\Sigma }}^{-1}\left(y-\bar{Y}\beta \right)\right]$$ (4)

Where

$$\alpha ={\left(2\pi \right)}^{-\mathrm{nT}/2}{\left|\Sigma \right|}^{-\frac{1}{2}}$$ (5)

Distribution of *β* is supposed to follow a normal multivariate distribution with mean $\beta_{0}$ and variance-covariance matrix ${\text{Ω}}_{0}$. In the original formulation by Dieppe et al. (2018) the expected value for each parameter (which contributes to the specification of the $\beta_{0}$ vector) is equal to 1 for the coefficients that multiply the variables in the first time lag (i.e., the time interval immediately before the one for which we aim to forecast the value, for example the day before in case of daily returns) and equal to 0 for the following lags, since most of the time series are characterized by the presence of a unit root. The variance-covariance matrix ${\text{Ω}}_{0}$ is a diagonal matrix whose terms are defined by a set of parameters usually derived from the econometric theory.

The chosen approach uses a slightly more complex variant of prior distribution compared to the Minnesota prior: the normal-inverse-Wishart prior. The main difference is related to the variance-covariance matrix of the errors Ʃ, which is also an unknown and is no longer known in advance, meaning that it has a stochastic behaviour (in line with the approach adopted in the Monte Carlo GARCH which also stochastically considers non-homoskedastic components). It is assumed that Ʃ follows an inverse Wishart distribution which has as input parameters the matrix Ω and the number of degrees of freedom v. In mathematical notation: $\Sigma_{WISHART}$∼$W^{-1}$ (Ω, *v*), where the matrix Ω is equal to the amount (v–number of parameters – 1) multiplied by the diagonal matrix that contains in the main diagonal the variance of the errors of each single variable calculated with AR models. For a discussion of the estimation of these parameters and other theoretical aspects linked to this econometrical model, we suggest the works of Karlsson (2012) and Giannone et al. (2015).

### Euler-Maruyama SDE numerical scheme with a EWMA volatility and a Cholesky Decomposition for the correlation

The Monte Carlo method in this context is interpreted as a numerical method that allows to simulate the possible trajectories of one or more assets that follow a Brownian geometric motion. A Brownian geometric motion is meant as a stochastic process defined by the SDE:

$$dS_{t}=\mu S_{t}\mathrm{dt}+\sigma S_{t}dW_{t}$$ (6)

Where μ is the mean of the asset returns, **σ** > 0 is the standard deviation represented by a EWMA approach with a smoothing factor equal to λ=0.94 (Haug, 2007), $S_{t}$ is the price of the asset at time t and $W_{t}$ is a Wiener process, that is a stochastic process defined by independent increments over time with mean equal to 0 and variance equal to the time interval considered: $W_{T}$ **-** $W_{0}$ is normally distributed with mean 0 and variance T.

In order to extend the model to a multi-asset portfolio it is necessary to take into consideration the correlation. We use the Cholesky decomposition in order to incorporate the correlation matrix for the four assets in the Monte Carlo simulation. Assuming you have a set of uncorrelated random numbers $\vec{\text{ε}}=\varepsilon_{1}$,$\;\varepsilon_{2}$,$\varepsilon_{3}$,$\ldots ..\varepsilon_{T}$, the Cholesky decomposition allows to transform them into a set of correlated variables $\vec{a}=$ $\;a_{1}$,$\;a_{2}$,$a_{3}$,$\ldots ..a_{T}$. If $\vec{\varepsilon }\;\text{and}\;\vec{a}$ are column vectors with $\varepsilon_{i}$ and $a_{i}$ in the rows, it is possible to transform $\vec{\text{ε}}$ to $\vec{a}$ by:

$$\vec{a}=M\vec{\varepsilon }$$ (7)

Where M is the matrix that must satisfy the condition $MM^{T}=R$, where R is a symmetric positive definite correlation matrix. M can be obtained by applying the Cholesky decomposition to R. Subsequently, the correlated shocks ($\vec{a}$) are substituted to the errors (**ε**). From this point, the various possible paths of the assets are simulated, thus obtaining a set of possible values of the returns from which to calculate ES with the quantile method. See Haug (2007) for a more detailed explanation.

With regards to the implementation of the model in the MATLAB^®^ environment, the Cholesky function has been used to transform the correlation matrix R into an upper triangular matrix M that would guarantee the respect of the condition $MM^{T}=R$. Subsequently, for each simulation, the Hadamard product has been used to multiply the innovations and the M matrix.

### Euler-Maruyama SDE numerical scheme with a GARCH volatility and a Cholesky Decomposition for the correlation

This approach inherits the structure from the previous approach and generalizes the volatility modelling using a GARCH process.

In a GARCH process (Engle, 1982), the conditional variance depends on the long-term unconditional volatility, the *p* most recent values of the variance and the square of the last *q* past returns, according to equation:

σt2=VLγ+ ∑i=1pαi ∗ ut−i  2+∑j=1qβj ∗ σt−j  2  (8)

Where $V_{L}$ is the unconditional (or long term) volatility, $u_{t-1\;}^{2}$is the squared log-return observed in t-1, and $\sigma_{t-1\;}^{2}$ is the conditional volatility observed in t-1. γ , $\alpha_{i}$ and $\beta_{j}$ are the three weights whose sum is equal to 1 (i=1,…,p and j=1,…,q). In the case of a GARCH(1,1) and assuming ω = $V_{L}\gamma$, the equation can be rewritten as:

$$\sigma_{t}^{2}=\omega +a*u_{t-1\;}^{2}+\beta *\sigma_{t-1\;}^{2}\;$$ (9)

By applying a Maximum Likelihood (ML) approach, it is possible to estimate the three parameters ω, α and β, obtaining then γ, where γ = 1-α-β. Writing the estimated variance in t as $v_{t\;}$ = $\sigma_{t}^{2}\;$and assuming that the probability distribution of *u* conditional to the variance $u_{t\;\;}^{2}$is normal, the ML equation that has to be maximized becomes:

L=∏i=1m12πvi exp(−ui22vi) (10)

By applying the natural logarithm and ignoring constant multiplicative factors, the previous equation can be rewritten as Hull (2014):

$$\mathrm{L=}\sum_{i=1}^{m}\left[-\ln\left(v_{i}\right)-\frac{u_{i}^{2}}{v_{i}})\right]=\sum_{i=1}^{m}L_{i}$$ (11)

The next step has a computational nature: by using a traditional numerical optimization, it is possible to obtain the value of the weights that maximize L. Once these weights have been estimated, we can insert the GARCH volatility in the Monte Carlo simulation that describes the dynamics of correlated assets with the Cholesky decomposition.

### Hybrid Monte Carlo Method That Uses the Predictions of NAR Network as Drift, a GARCH Volatility and a Cholesky Decomposition for the Correlation

In contrast to static neural networks, dynamic neural networks are characterized by the presence of feedback or delay at time t. Consequently, outputs at time t do not depend only on inputs, but also on outputs and state variables related to previous instants. Therefore, the dynamics are characterized by different memory levels and so neural networks, which can be trained with time dependent data, are suitable to be used as forecasting tools. NAR dynamic neural networks are widely used for forecasting; they are able to predict future values of a time series through the past values of the same series. If the time series is called y(t), it is possible to write: y(t) = f(y(t-1), y(t-2), …, y(t-n)), where the regressors y(t-1), y(t-2), …, y(t-n) are the past values of the time series.

**FIGURE 1:**
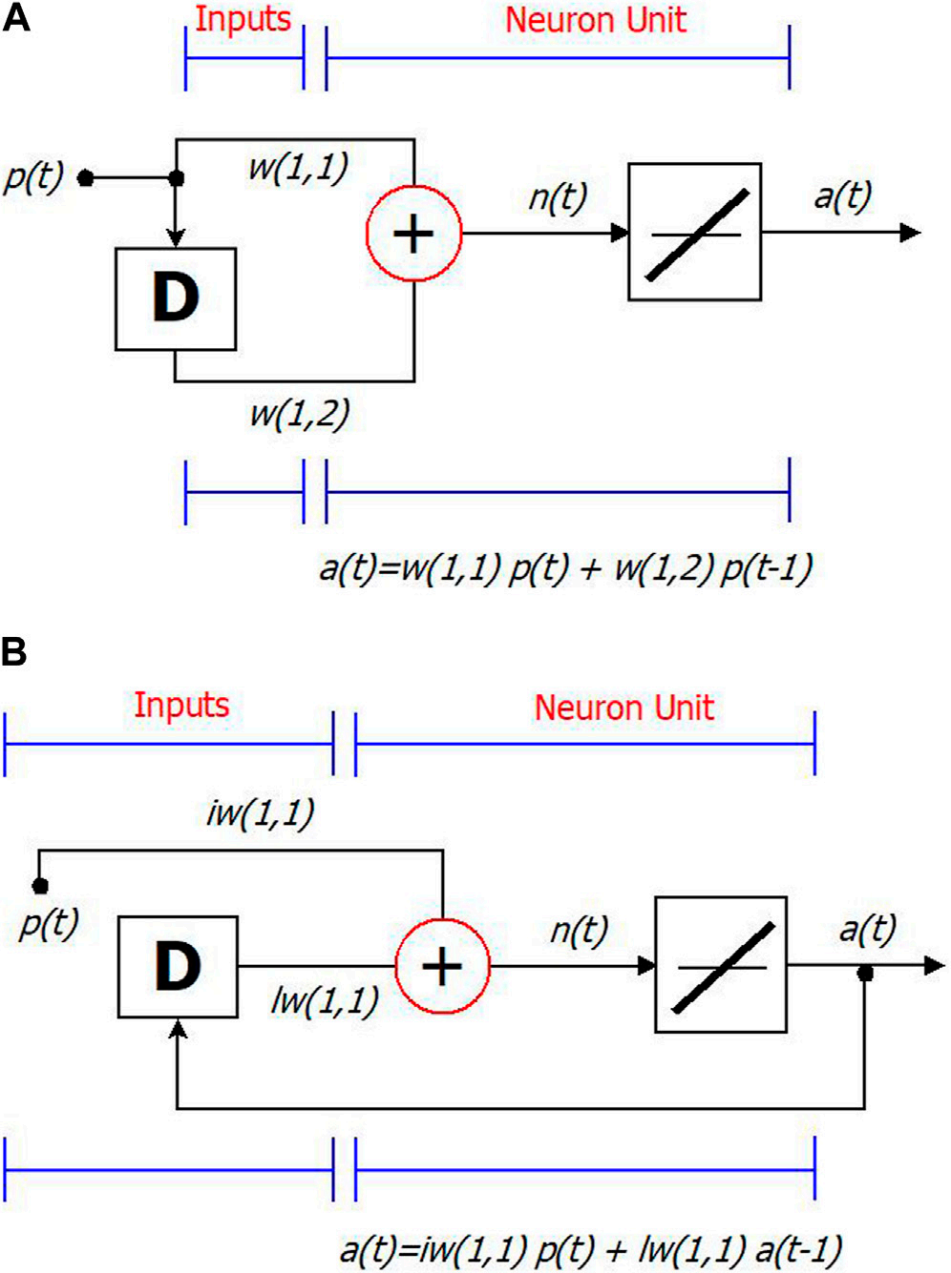
Feed-forward dynamic neural net with single-delay, single-layer and single-neuron **(A)**, recurrent dynamic neural net with single-delay, single-layer and single-neuron **(B)**.

Another important distinction within the class of dynamic neural networks is the one between feedforward and recurrent dynamic neural networks. In the former case, the memory of the network is limited to the delays on the input. Let us consider Figure 1A. In this case, the current network output a(t) depends on the current input p(t) weighted by w(1,1) and the previous input p(t-1) weighted by w(1,2), that is a(t) = w(1,1)∗p(t)+ w(1,2)∗p(t-1). Nevertheless, older inputs p(t-2), p(t-3),…, do not have an explicit influence on the network output; as a result, its memory is limited.

In the latter case, the presence of feedback connections makes the memory of the network theoretically limitless, let us consider the following Figure 1B.

In this case, the current network output a(t) is a function of the current input p(t) weighted by its input weight, iw(1,1) and the preceding network output a(t-1) weighted by its layer weight, lw(1,1), that is: a(t) = iw(1,1)*a(t-1)+lw(1,1)*a(t-1). In turn, a(t-1) is a function of p(t-1) and a(t-2), going backward in the chain, you obtain that the current network output a(t) is a function of all the input past values.

Extending these concepts to a more generalized recurrent architecture, we can say that a dynamic neural network is typically characterized by: a set of weight matrices (the number of which depends on the number of both network layers and delays) that can be associated with inputs or layers (IW and LW); a bias vector for each layer (b); a function combining inputs (or layer outputs) with biases, and usually expressed as a weighted sum for each neuron (adder block ⊕); a transfer function for each neuron (f); a variable number of delays, which can be applied to the inputs and/or the outputs of the layers. The outputs of Tapped Delayed Layers (TDL) can be reintroduced into the network in other layers.

Just as in static networks, the training phase is based on a gradient descent algorithm which calibrates the network parameters (IW, LW and b) in order to minimize a loss function (e.g. Mean Squared Error–MSE) which measures the error (estimated outputs vs. training values) observed during the training. The training phase of the net parameters is carried out taking into consideration all the best-practices used for having a reliable forecasting (Giribone, 2021). In particular, random splitting of the dataset is used to avoid overfitting and a double check of goodness of fitting and absence of error autocorrelation is carried out for each time prediction of the neural network.

## The Commitment Machine

A more precise ES estimation requires to selectively use the models described in the previous section. To this end, it has been decided to opt for an algorithmic solution, that we refer to as Commitment Machine (CM), which, given a set of ES methods considered statistically reliable, allows to select on each day t the method that best performed in the previous period and to use it in order to calculate the risk measure for the t+1 observation.

Given these specifications, the CM algorithm has been defined starting from three elements:

1) A set of methods for calculating the risk measures used, whose adequacy has been tested by the CM, together with the portfolio return data to be used for the back-testing and for the calculation of the loss function.
2) A loss function to be minimized that allows the metaheuristic to select the different calculation methods.
3) An observation window of n observations ranging from T- (n-1) to T which is used for estimating the loss function.

Regarding the first point, all methodologies are tested singularly on the portfolio and we obtain results that confirm the choice of including them in the basket of methods considered by the CM.

The second step needed in order to design the CM is the choice of the loss function to be minimized. Defining $R_{T}$ and $ES_{T}$ respectively as the returns at date T and the corresponding ES threshold, the possible loss functions are defined as:

$$\text{The}\;\text{ES}\;\text{threshold}\;\text{overruns}\;\text{function}\;\;\;f_{1}\left(R_{T}\right)=\{\begin{array}{c} 1,\;\;R_{T}\;<\;ES_{T}\\ 0,\;\;R_{T}\;\geq \;ES_{T} \end{array}$$ (12)

$$\text{The}\;\text{ES}\;\text{threshold}\;\text{loss}\;\text{function}\;\;\;f_{2}\left(R_{T}\right)=\{\;\begin{array}{c} \;{\text{ES }}_{T}-\;R_{T}\;,\;\;\;\;\;\;\;\;R_{T}\;<\;ES_{T}\\ 0,\;\;\;\;\;\;\;\;\;\;\;\;\;\;\;\;\;\;\;\;\;\;\;\;\;\;R_{T}\;\geq \;ES_{T} \end{array}$$ (13)

Minimizing the objective function, therefore, is equivalent to iteratively choosing the method of ES estimation which leads to lower ES losses as calculated in Eq. 13 or a lower overrun frequency as counted by Eq. 12. On average, for each method, we observed nearly one ES overrun every 30 market days, so Eq. 12 has a low sensibility. We therefore used Eq. 13 as the loss function to be minimized by the CM. These loss functions are very similar to the original ones proposed by Lopez (1998) and Sarma et al. (2003), however we use the Expected Shortfall as threshold instead of Value-At-Risk because it is a coherent risk measure.

The third step for the CM design is the determination of the time interval used for its calibration. This process defines the time interval where the metaheuristic selects the best method to use in the following step, according to the minimum loss functions. So, for example, if the CM has a time interval of 50 days, it will choose the method that has the lowest loss function calculated over the last 50 days.

In order to implement this calibration, we decided to test the algorithm over 11 possible observation windows of different lengths ranging between 50 and 100 days (50, 55, 60 .... 100 days) and to evaluate the ex-post performance of the CM for each observation window. For this case-study, we adopted a CM calibrated on an observation window of 55 days.

Among all the tested variants, the CM has the lowest overrun count (calculated as in Eq. 12) and a lower sum of losses (expressed for each method as the sum of the values estimated using Eq. 13 over the entire time series). Table 4 reports the values of the two loss functions for the four ES methods and the CM. The values of the loss functions for the other observation windows are very close to the optimum values obtained in Table 3; as a result, we can assert that the methodology is quite robust (Sum of ES losses varies between 50 and 54%, and ES overrun frequency spans from 2.21 to 2.48%).

**TABLE 4:** Overall value of loss function for the four ES methods and the CM.

	EWMA vol Monte Carlo	GARCH vol Monte Carlo	Bayesian method	NAR Monte Carlo	CM
Sum of ES losses	68,9%	76%	83,8%	88,1%	50,1%
ES overrun frequency (as % of total days)	4,01%	3,23%	3,01%	3,25%	2,21%

## Results

The analysis of the CM performances, tested on the realized returns of an equally weighted portfolio made up of four market indices from 2001 to 2020, shows that the use of the algorithm provides interesting advantages compared to the implementation of a single Expected Shortfall method. In particular, the analysis is divided into two steps: a disaggregated analysis of the performances of each CM on the days in which one of the four methods has been selected (NAR Monte Carlo ES, standard Monte Carlo with EWMA ES, standard Monte Carlo GARCH ES, Bayesian ES) and a general analysis of the performances of the CM over the entire dataset.

**FIGURE 2:**
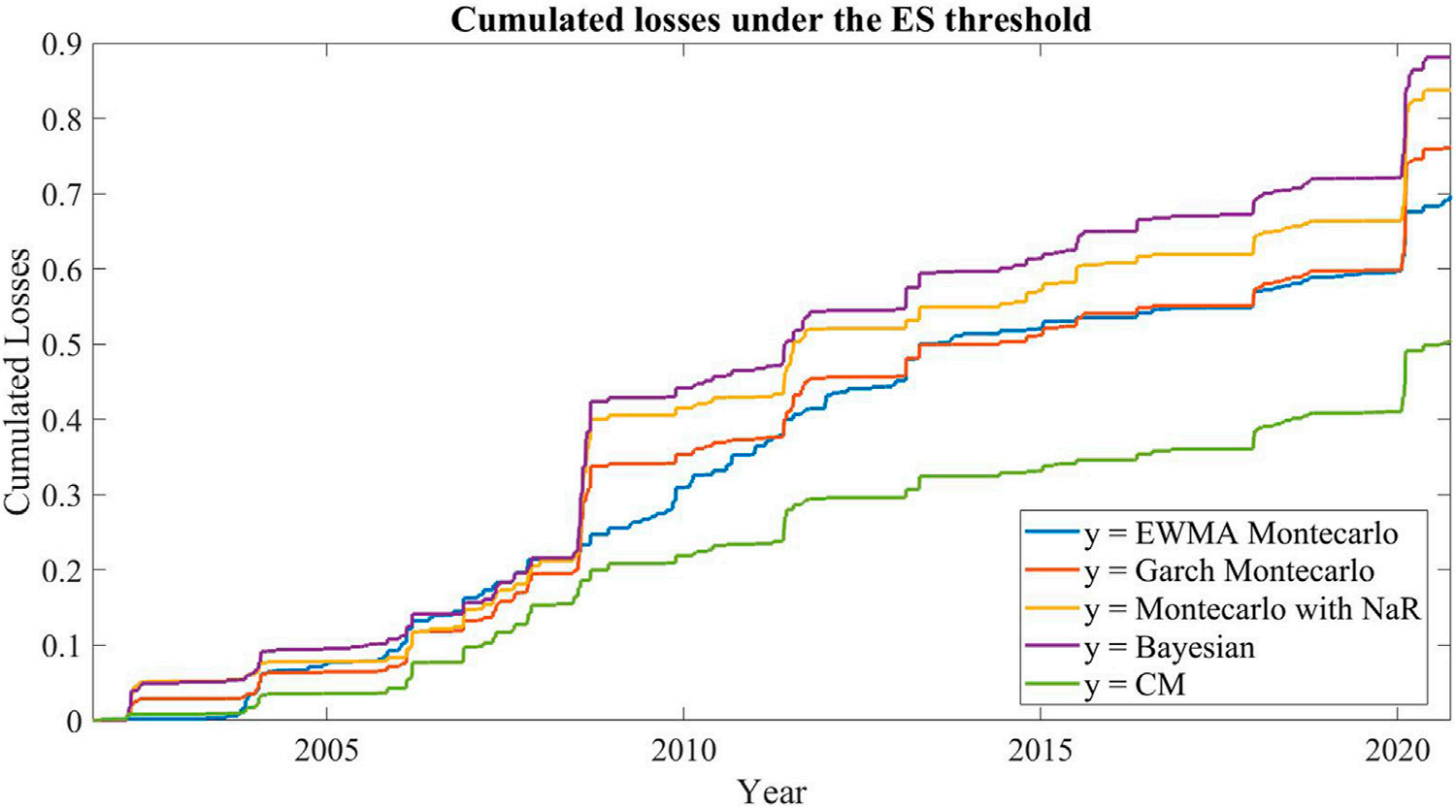
Cumulated losses below the ES threshold.

Thanks to its adaptive logic selection, the CM records lower ES violations and losses with respect to the four methods individually implemented, as shown in Table 4. Figure 2 shows, for the time interval considered, the cumulative losses with respect to the ES threshold of the four methods considered by the CM and of the two variants of CM. The cumulative loss on a certain date t is equal to the sum of the historical values from 0 to t of the loss function specified in *The Commitment Machine* in Eq. 13 and it represents the total amount of losses below the ES threshold up to that day.

Data show two periods in which the portfolio faces large losses, specifically the subprime mortgage crisis and the Covid shock in the spring of 2020. Between these two periods there is a further period of losses–of smaller magnitude compared to the other two - corresponding to the crisis of the sovereign debts in the Eurozone. During these two negative market events, the cumulative losses increased considerably for the four ES methods used and far less for the CM based on these methods.

Overall, the CM algorithm suffers far fewer losses than the other four methods, particularly during the two periods of market crisis. This is a further endorsement of the validity of the heuristic approach adopted in the design phase, which prevents the algorithm from selecting those methods that have the worst performance.

To better contextualize these results, it is important to evaluate the relative performances of all the ES calculation methods. To this end, we proceed to further break down the analysis and we consider, for each CM, the days in which each ES method has been selected. The dataset has been divided into four sets, each containing the days in which one of the four methods has been selected. Then, the average losses below the ES threshold (that is, the sum of values assumed by Eq. 13 divided by the number of days in which each method is selected) have been calculated for each method in each of the four sets, and these data have been compared with the performances of the other methods on the same days. For example, a value of 0.1% means that on average we have a 0.1% negative difference between ES threshold and market percentage return.

Table 5 shows, for each method in each of the four sets, the average values of Eq. 13 that computes total losses below the ES threshold, expressed as the mean of total % value as explained in the previous paragraph.

**TABLE 5:** Comparative average of losses below the ES threshold.

	Average ES threshold loss when the CM selects one of these methods			
	EWMA vol Monte Carlo	GARCH vol Monte Carlo	NAR Monte Carlo	Bayesian method
EWMA vol Monte Carlo	**0,020%**	0,042%	0,048%	0,052%
GARCH vol Monte Carlo	0,027%	**0,017%**	0,018%	0,018%
NAR Monte Carlo	0,024%	0,017%	**0,017%**	0,017%
Bayesian method	0,027%	0,019%	0,019%	**0,017%**

Performances of the individual methods on the days in which they have been selected are highlighted in bold.

For example, the first row of Table 5 shows, for each ES estimation method, the average amount of losses of the Expected Shortfall threshold calculated on the days in which the method selected is the EWMA volatility Monte Carlo ES. It is expected that, on the days in which the CM chooses this method, this figure will be lower for the EWMA volatility Monte Carlo ES. This means that, when it is chosen, the EWMA is the best method: the CM is able to choose, in T+1, the method with the smallest loss below the ES threshold.

In order to simplify the analysis, performances of the individual methods on the days in which they have been selected are highlighted in bold. For each row, these performances are expected to be the best. The analysis of the results shows a significant selection ability of the CM: in all the considered cases, the CM is able to manage the choice of the method which guarantees the smallest losses. This is an important confirmation of the statistical validity of our approach,

Relatively less brilliant performances for the Monte Carlo method that takes into account the NAR are observed; however, when selected, this method outperforms the more traditional Monte Carlo approaches based on EWMA. It also outperforms the GARCH Monte Carlo and Bayesian method, but by a very small quantity. When selected, the EWMA is the method that has the greatest advantage in terms of performances if compared with the other ones. As we will see, it is selected during the moments of financial crisis because of its very prudential representation of the short-term volatility.

Table 6 reports the same analysis as Table 5 but for the frequency of the overruns of the ES threshold calculated as reported in Eq. 12, instead of average losses below the ES threshold. The results confirm the analysis presented in this paragraph: the CM appears to provide a robust metaheuristic in relation to the ability to select, day by day, a method that provides a reliable ES selection by estimating a threshold that is less likely to be violated. The only exception is the Monte Carlo GARCH method, that has strictly larger losses than the Bayesian method (0.024 vs. 0.023), when selected.

**TABLE 6:** Comparative average of overruns below the ES threshold.

	Average frequency of ES threshold overrun when the CM selects one of these methods			
	EWMA vol Monte Carlo	GARCH vol Monte Carlo	NAR Monte Carlo	Bayesian method
EWMA vol Monte Carlo	**0,021%**	0,041%	0,040%	0,045%
GARCH vol Monte Carlo	0,05%	**0,024%**	0,027%	0,023%
NAR Monte Carlo	0,045%	0,028%	**0,024%**	0,027%
Bayesian method	0,048%	0,030%	0,030%	**0,022%**

Performances of the individual methods on the days in which they have been selected are highlighted in bold.

**FIGURE 3:**
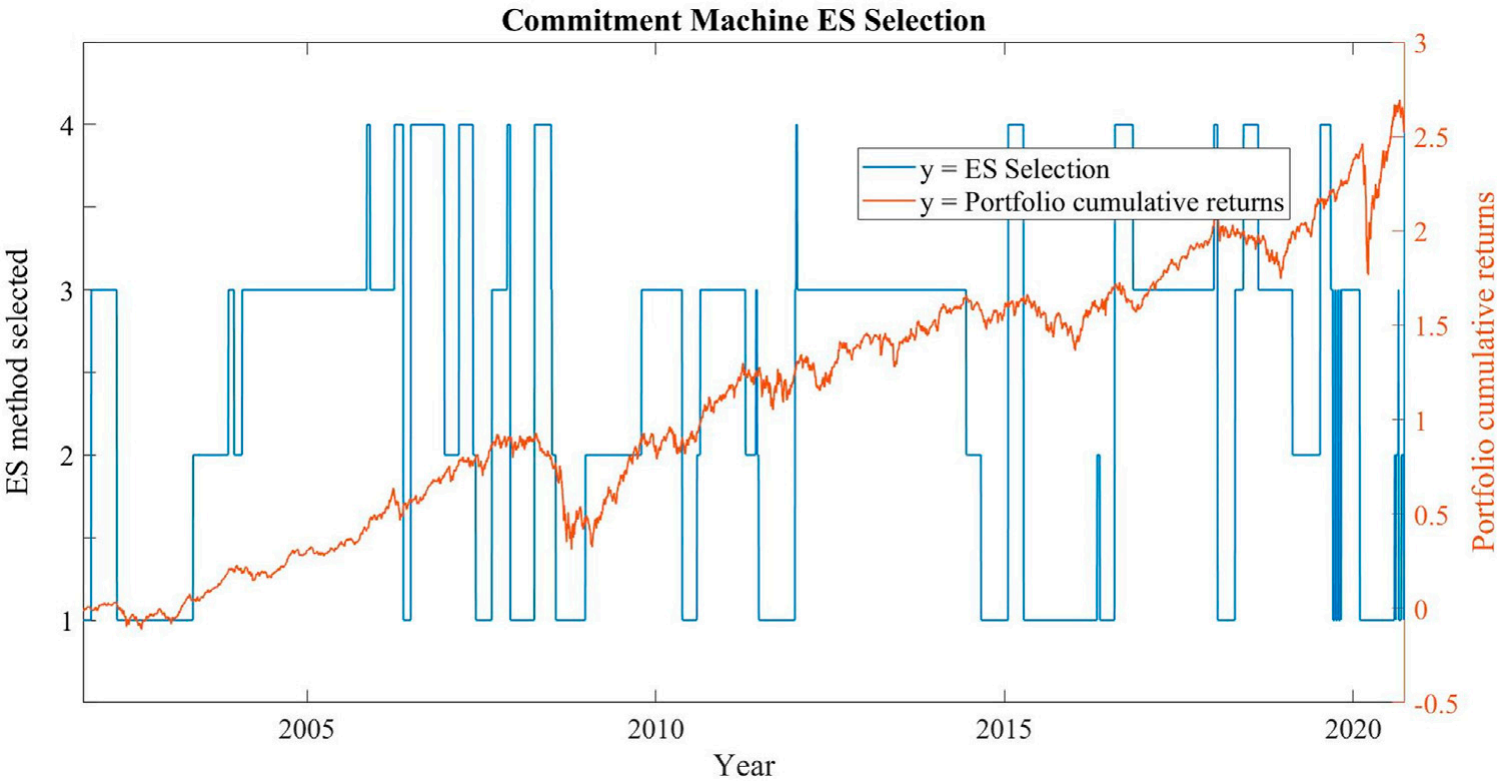
Commitment machine ES selection.

Another important feature of our CM is the stability of the model. Figure 3 shows the type of ES model selected by the algorithm (1 = standard Monte Carlo with EWMA, 2 = standard Monte Carlo with GARCH, 3 = Bayesian method, 4 = Monte Carlo with NAR) and the performance of the portfolio (measured by cumulative returns) from the beginning of the dataset to the end.

Observing Figure 3, it can be noticed that the method chosen by the CM remains the same for long periods. This is a very important factor, as it highlights how the CM's choices are consistent over time. There is no random selection, but a logical choice. In fact, for 98.6% of the days of our dataset, the method chosen by the CM in that day is the same method chosen in the previous market day.

It is worth noting that there are three different periods (2004–2006, 2013–2014, 2017) in which the CM selects the Bayesian method. These periods are characterized by low volatility and (with the exception of 2017) relatively low correlation coefficients between the two equity indexes. It is reasonable to assume that in these conditions the normal-inverse-Wishart prior described in paragraph 3.1 can generate a more prudential forecast, because it includes in its simulation the possibility of a growth of both correlation and volatility.

The periods of greater instability–in terms of CM selection–are the 2 years leading to the two market crises of subprime bonds and covid pandemic. Both these periods of growing cross-correlations between the two stock market indexes end up with a sudden market crash in which we observe $\sigma_{short\;term\;\;}^{t}$>$\;\sigma_{long\;term\;\;}^{t}.$ In our framework, a long term window consists of 260 days and covers all the period for which each risk measure is calibrated (before the loss function calculation), while the short term window is 50 days long and describes the short term behaviour of the market more accurately. In this context, a EWMA is a more prudential representation of market conditions, because it gives very little weight to long term volatility, far less than the GARCH representation.

More in general, correlation plays an important indirect role in determining the CM performances and in order to analyse this issue, we created a “reduced” CM machine variant, that considers only the standard Monte Carlo representation with EWMA and GARCH Volatility (that we will call CM2) with an observation window of 55 days.

For each day, we calculated the difference between the cross correlation between the two stock indexes computed over 55 days (the observation window) and the same coefficient calculated over 260 days (the calibration period). This difference explains how the correlation changes over time: a positive difference means that we are in a period of rising market cross correlations, while a negative difference means that we are in a period of declining cross correlations. Also, if the difference is positive, this means that the CM is working on a period of 55 days with high correlation, and this correlation was lower in the previous period of 260 days. The ES methods are likely suffering from the fact that they were calibrated on a less correlated sample, one in which the risk associated with market correlation was smaller. The opposite holds if the difference is negative.

For the CM2, we find out that the EWMA variant is selected in days with an average correlation coefficient difference of +0.032, while the GARCH variant is selected in days with an average correlation coefficient difference of −0.031. On average, the EWMA is selected in periods in which the correlation is increasing, while the opposite holds for the GARCH variants. The fact that, on average, the short-term volatility representation of EWMA is more prudential (and thus better than the GARCH) in periods in which correlation increases is related to such increase being associated with periods of market instability and larger short-term volatility. On the contrary, periods of decreasing correlation are relatively stable in terms of return and volatility: in such periods, it is more prudent to give a larger weight to long term volatility, that may represent periods of bigger negative co-movements between assets.

If we look at the same data as used with the original commitment machine that considers all of the four methods, the average correlation difference for the days in which each method is selected is positive for standard Monte Carlo with EWMA and negative for all the other methods.

**FIGURE 4:**
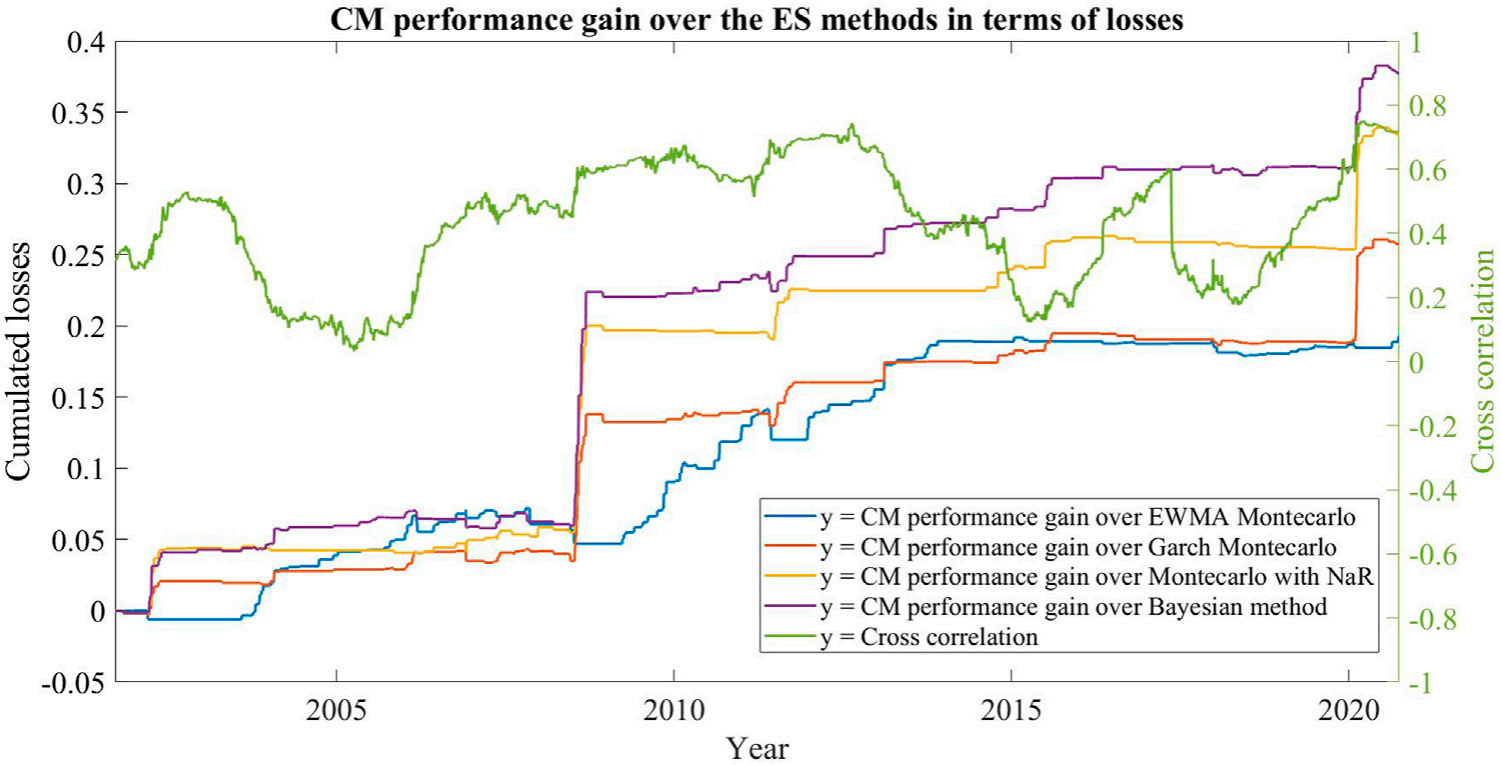
Commitment machine performances and asset cross-correlations.

Figure 4 shows the difference between the cumulated losses of the four methods and the losses of the CM during periods characterized by different cross correlation of the two equity indexes. Such difference can be interpreted as the relative performance of the CM with respect to the original four methods and it turns to be bigger during market crashes (the two spikes on the plot correspond to the 2008 and the 2020 crises) when correlation between assets grows and all the portfolio components start to move in the same downward direction. These periods of rising correlations can be interpreted as moments of change in the market behaviour, when the adaptive selection of the CM has a distinct advantage over the individual ES estimation methods: as the market conditions change, so does the model used by the CM.

This pattern confirms that the CM machine is a reliable tool for Risk Management during periods of instability and helps to define a first context in which our CM can be effectively used.

## Conclusion

Our analysis provides evidence in favor of the goodness of the design of the proposed CM, thus making it a useful tool for managing portfolio risk. In this paper we have designed an algorithm which performs an automatic choice among different Expected Shortfall methods, based on the minimization of a loss function that takes into account the negative returns below the ES threshold.

The proposed solution consists in the implementation of a metaheuristic, called Commitment Machine (CM), that allows to choose, day by day, the ES estimation that reasonably fits the financial market conditions best. In accordance with the important role that correlation plays in the financial contagion and systemic risk, we have decided to employ four ES estimation models that are able to incorporate this fundamental factor in the simulation:

A) a Bayesian Vector Autoregressive model (BVAR).
B) a Euler-Maruyama SDE numerical scheme with a EWMA volatility and a Cholesky Decomposition for the correlation.
C) a Euler-Maruyama SDE numerical scheme with a GARCH volatility and a Cholesky Decomposition for the correlation.
D) a hybrid Monte Carlo method that uses the predictions of Non-linear Autoregressive (NAR) networks as drift, a GARCH volatility and a Cholesky Decomposition for the correlation.

These different models are able to take into account different econometric aspects of the time series, particularly non-stationarity and cross correlation. We tested the analysis of the CM performances on the realized returns of an equally weighted portfolio made up of four market indices from 2001 to 2020. Thanks to its adaptive logic selection, the CM records lower ES violations and losses than the four methods individually implemented.

Moreover, the flexibility of the code written in MATLAB^®^ environment guarantees the possibility of generalizing the analysis by including other ES estimation methods, other than the ones used in this work. Finally, the proposed algorithm can be easily extended, for example by modifying the loss function in order to consider the needs of the various entities involved in the risk assessment or by including a more realistic trade-off between excess losses and the opportunity cost related to a too prudential ES threshold. As shown in Bagnato et al. (2021), it is also possible to consider any risk measure other than the ES and use it to calculate our loss function.

In conclusion, our applied analysis provides significant evidence in favor of the goodness of the design of the proposed CM, thus making it a useful tool for managing portfolio risk.

## Data Availability

The raw data supporting the conclusions of this article will be made available by the authors, without undue reservation.
